# Psychometric Properties of the Arabic Version of the Mini-IPIP Five-Factor Personality Scale in Kuwait

**DOI:** 10.1192/j.eurpsy.2025.1902

**Published:** 2025-08-26

**Authors:** B. M. Alansari

**Affiliations:** 1 Department of Psychology, University of Kuwait, Shuwaikh, Kuwait

## Abstract

**Introduction:**

The Mini-IPIP, a 20-item short form of the 50-item International Personality Item Pool-Five-Factor Model measure (Goldberg, 1999), with promising psychometric properties, The Mini-IPIP describes personality in terms of the Five Factor Model, namely Neuroticism, Extraversion, Openness to Experience, Agreeableness, and Conscientiousness. The Mini-IPIP appears to be an alternative to the Ten-Item Personality Inventory (TIPI), and the Big Five Inventory (BFI).

**Objectives:**

The study aims to investigate the psychometric properties of the Arabic version of the Mini-IPIP.

**Methods:**

The Arabic version of the Mini-IPIP scales, and the TIPI was administered to1560 Kuwait university undergraduates (576 males mean age = 21.82±0.70 and 984 females; mean age = 20.95±1.31). The internal consistency reliability, factor structure, and convergent validity of the Mini-IPIP with TIPI & BFI were assessed.

**Results:**

Cronbach’s alpha was satisfactory for N (0.82), E (0.73), O (0.75), A (0.81) and C (0.80). Results revealed significant gender differences in N with a favor for females and in E, O & C with a favor for males. PCA showed that Mini-IPIP five factors explains 71.05 % of the total variance. The Mini-IPIP demonstrated good convergent validity with the BFI (r =71, .76, .77, .82, .67 for E, A, C, N, and O, respectively) and with the TIPI (r = .46, .49, .66, .69, .58 for E, A, C, N, and O, respectively).

**Conclusions:**

The findings support the psychometric properties of the Arabic version of the Mini-IPIP as a useful tool for researchers needing a short assessment of the Big Five in research.

**Disclosure of Interest:**

None Declared

